# Bile Chemistry During Ex Situ Normothermic Liver Perfusion Does Not Always Predict Cholangiopathy

**DOI:** 10.1097/TP.0000000000005244

**Published:** 2024-12-07

**Authors:** Christopher John Edward Watson

**Affiliations:** 1 Department of Surgery, University of Cambridge School of Clinical Medicine, Cambridge, United Kingdom.

On behalf of my coauthors, I would like to thank Miceli et al for their interest in our article, their appraisal of it, and their comments. I address the points they raise as follows:

1. Miceli et al reiterated our concerns regarding the use of bile analytes to assess viability. Although they are useful, they are not infallible; the concentration of analyte in the perfusate can strongly influence the concentration in the bile, especially for analytes such as pH and glucose where there are powerful gradients created by ion pumps on the cholangiocyte membrane, which may become saturated.
2. Miceli et al suggested that bile-perfusate ratios may better capture cholangiocyte function. This is not correct. First, some analytes, such as bile pH, can exceed the measurement abilities of most point-of-care analyzers. Second, ratios fail when pumps saturate and can no longer excrete or reabsorb analytes from the bile duct lumen. This is best illustrated with glucose, as illustrated in Figure 4 of our article. Assuming normal function and that the glucose pump reabsorbs all glucose up to a gradient of 10 mmol/L, such that for any perfusate glucose above 10 mmol/L there will be [P]-[B] mmol/L glucose remaining in the bile (see Table 1). As the perfusate glucose falls below 10 mmol/L, glucose remains as a trace in the bile, typically around 1 mmol/L. The bile:perfusate ratios change from decreasing as the perfusate glucose falls to increasing once the perfusate glucose concentration is less than the capacity of the glucose pump to reabsorb glucose (see Table 1), and also livers B, E, F, and J of Figure 5 in the original article.^1^ Matton et al^2^ also recommended ratio thresholds for glucose, which we demonstrated in our article to be unreliable.
3. We acknowledge that nonanastomotic strictures of the bile duct may have multiple causes, but our evidence points strongly toward a “microthrombotic” origin as a significant cause. This is based on the association of high perfusate D-dimer levels in livers developing cholangiopathy, as well as histological evidence of fibrin plugs associated with necrotic stroma.^3,4^ In addition, we have pilot data suggesting that treatment with tissue plasminogen activator during normothermic machine perfusion minimizes the incidence of cholangiopathy, and this has prompted us to initiate a multicenter randomized controlled trial of this treatment.
4. Miceli et al cited their article looking at biopsies of a subset of livers in the COPE study.^5,6^ These were biopsies of the terminal end of the bile duct. Given the trauma associated with cannulation of the common duct both at organ recovery and the presence of the cannula during perfusion, we do not consider these biopsies to be representative of intrahepatic bile duct morphology. Indeed, we failed to find a correlation between common duct histology and the histology of second-order and third-order bile ducts in a series of livers perfused for research. It was thus no surprise that no correlation was found between bile duct injury score and the occurrence of posttransplant cholangiopathy in their cohort. Bile chemistry was not studied in the COPE study.
5. Miceli et al recommended looking at the temporal evolution of bile:perfusate ratios rather than single time-point assessments or threshold values. Our article demonstrated the temporal evolution of individual analytes and also provided bile:perfusate ratios. The reader may decide for themselves which they find the most useful. Based on >400 liver perfusions to date, we would prefer to study the evolution of the absolute analyte values of bile and perfusate pH and glucose during perfusion.
6. Miceli et al suggested that our study reporting bile chemistry of 200 livers showed that “livers that did not develop non- anastomotic stricture maintained a constant bile:perfusate pH ratio during normothermic machine perfusion.” This is not correct and is a misinterpretation of a graph showing median and interquartile ranges of pH values for 200 livers. Within those values, individual livers may have had a rising pH and some a falling pH ratio. In addition, one of the problems in quoting a bile:perfusate pH ratio, which we mentioned in our article, is that the point-of-care analyzers may not be able to measure the upper extent of the bile pH. If the pH exceeds the upper measurement threshold of, say, 7.8, the ratio either cannot be quoted or must be defaulted to one based on an incorrect bile pH of 7.8, and thus the ratio is artificially lowered. This is a technical issue and not necessarily a portent of good or bad cholangiocytes.
7. Finally, we concur with the authors that large registries are required to record analyte concentrations at the same time points to better estimate the likelihood of cholangiopathy. With respect to hepatocyte viability, this will, in part, reflect the function of the liver during perfusion and will, in part, reflect the recipient into whom it is placed—a poorly functioning liver undergoing normothermic perfusion may recover good function in a fit recipient but fail if implanted into a sick recipient having an extreme intraoperative hemorrhage.

**TABLE 1.** Example of a liver in which the cholangiocyte glucose pump saturates at a bile:perfusate difference of 10 mmol/L and its effect on the bile:perfusate glucose ratios

Perfusate concentration, mmol/L	Bile concentration, mmol/L	Perfusate:bile concentration difference, mmol/L	Bile:perfusate glucose concentration ratio
30	20	10	0.67
20	10	10	0.50
15	5	10	0.33
10	1	9	0.10
5	1	4	0.20
